# Targeted de-repression of neuronal Nrf2 inhibits α-synuclein accumulation

**DOI:** 10.1038/s41419-021-03507-z

**Published:** 2021-02-26

**Authors:** Paul S. Baxter, Nóra M. Márkus, Owen Dando, Xin He, Bashayer R. Al-Mubarak, Jing Qiu, Giles E. Hardingham

**Affiliations:** 1 UK Dementia Research Institute at the University of Edinburgh, Edinburgh Medical School, University of Edinburgh, Edinburgh, UK; 2 Deanery of Biomedical Sciences, Edinburgh Medical School, University of Edinburgh, Edinburgh, UK; 3 Simons Initiative for the Developing Brain, Edinburgh Medical School, University of Edinburgh, Edinburgh, EH8 9XD UK

**Keywords:** Cellular neuroscience, Parkinson's disease, Astrocyte, Molecular neuroscience

## Abstract

Many neurodegenerative diseases are associated with neuronal misfolded protein accumulation, indicating a need for proteostasis-promoting strategies. Here we show that de-repressing the transcription factor Nrf2, epigenetically shut-off in early neuronal development, can prevent protein aggregate accumulation. Using a paradigm of α-synuclein accumulation and clearance, we find that the classical electrophilic Nrf2 activator tBHQ promotes endogenous Nrf2-dependent α-synuclein clearance in astrocytes, but not cortical neurons, which mount no Nrf2-dependent transcriptional response. Moreover, due to neuronal Nrf2 shut-off and consequent weak antioxidant defences, electrophilic tBHQ actually induces oxidative neurotoxicity, via Nrf2-independent *Jun* induction. However, we find that epigenetic de-repression of neuronal Nrf2 enables them to respond to Nrf2 activators to drive α-synuclein clearance. Moreover, activation of neuronal Nrf2 expression using gRNA-targeted dCas9-based transcriptional activation complexes is sufficient to trigger Nrf2-dependent α-synuclein clearance. Thus, targeting reversal of the developmental shut-off of Nrf2 in forebrain neurons may alter neurodegenerative disease trajectory by boosting proteostasis.

## Introduction

The transcription factor Nrf2 is a widely expressed stress-responsive regulator of several aspects of homoeostatic physiology. Under basal conditions it is held in the cytoplasm and targeted for ubiquitin-mediated degradation by its inhibitor Keap1. However, in response to signals, including oxidative stress and heavy metal toxicity, its interaction with Keap1 is altered, preventing degradation, and allowing it to translocate to the nucleus^1^. Here, it activates expression of a battery of genes containing antioxidant response elements in their promoter, including antioxidant pathway genes, and xenobiotic detoxification genes^1–4^.

Importantly, Nrf2 has also recently been found to be capable of regulating proteostasis, both directly and indirectly, through a number of gain- and loss-of-function studies. Nrf2 activity can be manipulated by conventional over-expression or knock-down, and endogenous Nrf2 dependent gene expression can also be activated by disrupting the ability of Keap1 to promote Nrf2 degradation, using a number of small molecules, most commonly acting via the electrophilic modification of redox sensitive Keap1 cysteine residues^1,5,6^. This pharmacological activation approach has been shown in pancreatic ß-cells, mouse embryonic fibroblasts and breast cancer cells to repress cytotoxicity and unfolded protein response (UPR) over-activation in response to ER stress inducers, at least in part through maintenance of ER redox balance and disulphide chemistry^7–9^. In addition to modulating the UPR, Nrf2 controls other key arms of cellular proteostasis machinery. In liver cells, fibroblasts and human ES cells, Nrf2 has been shown to control capacity of the ubiquitin proteasome system (UPS). In addition, Nrf2 boosts the macroautophagy pathway, as evidenced from studies in HeLa cells and MEFs^10,11^.

In the brain, many neurodegenerative diseases are associated with neuronal protein aggregate accumulation, such as α-synuclein (Parkinson’s disease, Lewy Body dementia), TDP-43 (motor neuron disease, fronto-temporal dementia (FTD)) and Tau (Alzheimer’s disease, FTD), and both genetics, molecular pathology and animal studies point to defects in proteostasis in patho-progression^12–14^. At first glance, this suggests that small molecule activators of endogenous Nrf2 could be employed to limit aggregate accumulation by controlling proteostasis machinery via mechanisms such as those described above in non-neuronal cells. However, forebrain neurons are highly unusual in that they express very low levels of Nrf2, due to epigenetic repression of the Nrf2 promoter early in development, and the little Nrf2 that remains is highly unstable^15–17^, suggesting that endogenous neuronal Nrf2 may be insufficient to support a proteostatic response, and that novel strategies may be required to overcome the transcriptional repression of this key cytoprotective gene. Here we have devised novel strategies to reverse neuronal Nrf2 repression, and consequently drive endogenous Nrf2-dependent α-synuclein clearance.

## Results

### Activation of endogenous Nrf2 can drive α-synuclein clearance in astrocytes but not neurons

To establish whether activators of endogenous neuronal Nrf2 could promote protein aggregate clearance in neurons, we employed a model of cortical neuronal α-synuclein accumulation, a core pathological signature in Lewy Body Dementia. We chose this model as it had been recently shown that ectopic over-expression of Nrf2 in cortical neurons could modulate proteostasis and promote α-synuclein clearance^18^. Thus, while this approach drives Nrf2 to unphysiological levels, the study did reveal that neurons possessed the downstream machinery to clear α-synuclein in response to elevated Nrf2.

Mixed neuron/astrocyte cortical cultures (~10% astrocytes) were transfected with an α-synuclein-encoding vector. We observed that the quantity of α-synuclein plasmid, and the level of α-synuclein expression detected (by immunofluorescence) was approximately linearly related, confirmation that the detection approach was quantitative and able to reveal increases or decreases in expression (Fig. S1a,b). The expression of α-synuclein was cell-wide, as is known to be the case when α-synuclein is pathologically over-expressed, such as in the Thy1- αSYN mouse model, or in human Parkinson’s disease patients with *SNCA* gene triplication^19–22^. In these α-synuclein expressing cells, instead of over-expressing Nrf2^18^, cells were treated with the electrophilic Nrf2 activator tBHQ for 24 h, after which accumulated α-synuclein was quantified (blind) in both neurons and astrocytes.

**Fig. 1:**
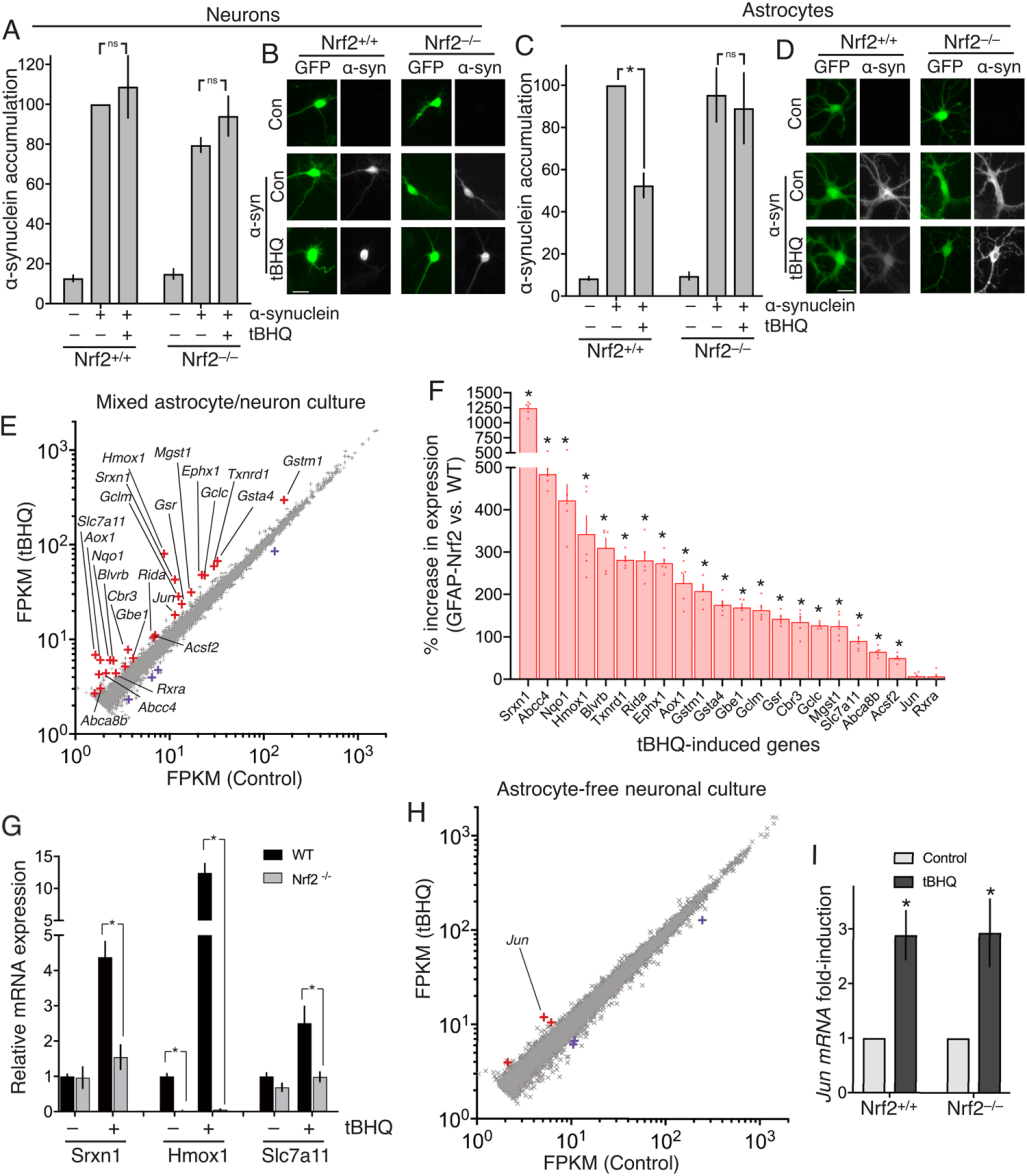
Endogenous Nrf2 cannot clear α-synuclein or support an Nrf2-dependent transcriptional response in neurons, unlike astrocytes. DIV10 (Days In Vitro) mixed neuron/astrocyte cultures were transfected on DIV3 with vectors encoding eGFP, plus either α-synuclein (pCAGS- α-synuclein) or a ß-globin control. 5 days post-transfection, cells were treated ± tBHQ (10 µM) for 24 h, fixed, and subjected to immunofluorescence cytochemistry (anti- α-synuclein antibody) (BD Transduction Laboratories, #610878 (1:1000)). Immunofluorescence images were taken and quantified (blind). Neurons and astrocytes were distinguished morphologically (astrocytes have larger nuclei, thick proximal processes, and an absence of axons), a method that was validated separately using neuronal (NeuN) and astrocytic (GFAP) markers. **A, B** Analysis and example pictures of neurons. 43-77 cells per condition, *n* = 4 independent experiments. Scale bar: 25 µm. **C, D** Analysis and example pictures of astrocytes. *P = 0.004 2-way ANOVA plus Tukey’s post-hoc test; *n* = 4 independent experiments (22–30 cells analysed for each condition). Scale bar: 25 µm. **E** DIV10 mixed neuron/astrocyte cultures^17^ were treated ± tBHQ (10 µM,) for 8 h, RNA extracted and subjected to RNA-seq analysis (ca. 35 × 10^6^ paired-end reads/sample, 3 biological replicates; EBI ref: E-MTAB-5688). Mean data for the 11,698 genes expressed on average >2 FPKM is plotted. Differentially expressed genes (DESeq2 v1.6.3, Benjamini-Hochberg-adjusted *P* value < 0.05) are highlighted red (induced) or blue (repressed). Genes induced >1.5-fold are labelled. **F** Genes labelled in red in 1e were cross referenced to RNA-seq data from astrocytes over-expressing Nrf2 (FAC-sorted from the GFAP-Nrf2 mouse). Of the 22 genes that were expressed in this GFAP-Nrf2 RNA-seq data set >2 FPKM, the % difference in GFAP-Nrf2 astrocytes (vs. WT) is shown. *(p_adj<0.01, *n* = 5, see Supplemental Table S1 for exact *p* values). **G** qPCR analysis of the indicated genes in RNA extracted from mixed cultures of the indicated genotypes, treated with tBHQ as in 1e. * *P* value < 0.05 *t* test, *n* = 3–5 per condition. **H** Experiment performed as in (**E**) except that astrocyte-free neuronal cultures^17^ were employed. All 10,845 genes expressed on average >2 FPKM are plotted, and differentially expressed genes (DESeq2 v1.6.3, Benjamini-Hochberg-adjusted *P* value < 0.05) are highlighted red (induced) or blue (repressed). **I** Astrocyte-free Nrf2^+/+^ or Nrf2^–/–^ cortical neurons were treated as in (**F**) and *Jun* mRNA expression analysed (by qPCR). *P = 0.005, 0.004 (reading left-to-right here and throughout the manuscript), 1-way ANOVA plus Sidak’s post-hoc test (*n* = 6).

We analysed both cell types since astrocytes express higher levels of Nrf2 than neurons^15,17^ and we therefore hypothesized that astrocytes would respond more robustly to tBHQ than neurons. We found that tBHQ treatment (10 µM) significantly reduced α-synuclein accumulation in astrocytes, but failed to reduce α-synuclein in cortical neurons (Fig. 1a–d). We confirmed that this effect of tBHQ in astrocytes was mediated by Nrf2: tBHQ was found to have no effect on α-synuclein in astrocytes derived from Nrf2^–/–^ mice (Fig. 1c,d). Thus, α-synuclein clearance induced by endogenous Nrf2 activation can be achieved by tBHQ treatment of astrocytes but not neurons. It is important to note that these experiments are simply a proof-of-concept that Nrf2 activation has the potential to clear α-synuclein in cells where Nrf2 is activatable.

### tBHQ induces Nrf2-independent induction of Jun and neurotoxicity

To investigate the transcriptional responses to tBHQ associated with these marked differences in α-synuclein clearance capacity in neurons vs astrocytes, we first performed RNA-seq on the mixed astrocyte-neuron cultures (described above) treated ± tBHQ (10 µM) and observed transcriptional induction of 25 genes (> 2 FPKM, > 1.5-fold, P_adj < 0.05, Fig. 1e, Supplemental Table S1). While this list contains many known Nrf2 target genes, we wanted to determine whether any non Nrf2-mediated responses were apparent. We cross-referenced these genes to RNA-seq data we have obtained from astrocytes over-expressing Nrf2 (FAC-sorted from the GFAP-Nrf2 mouse^23^). Of the 22 genes that were expressed in this GFAP-Nrf2 RNA-seq data set >2 FPKM, 20 are significantly up-regulated in GFAP-Nrf2 astrocytes, relative to wild-type (p_adj<0.01, *n* = 5, Fig. 1f, Supplementary Table S1). Moreover, there exists independent published evidence that all of the 20 genes are direct Nrf2 targets^24–28^. We also confirmed that the tBHQ-induced expression of a selection of genes- *Hmox1*, *Srxn1*, and *Slc7a11*, was strongly abrogated in Nrf2-deficient cultures (Fig. 1g). Of note, the tBHQ-induced gene *Jun* was not significantly up-regulated in GFAP-Nrf2 astrocytes, indicative of Nrf2-independent induction, and in agreement with the absence of published evidence that *Jun* is an Nrf2 target gene.

We had previously shown using analysis of selected Nrf2 target genes, that Nrf2 target gene expression in mixed cultures is due to the astrocytic response, not the neuronal one^17^. We therefore performed RNA-seq on astrocyte-free neuronal cultures treated ± tBHQ (10 µM) and found that strikingly, no known Nrf2 target genes were induced at all (Fig. 1h). A single gene was induced >2-fold: the AP-1 family member and stress-response gene *Jun* (Fig. 1h, Supplementary Table S2). *Jun* is not a known Nrf2 response gene, and (as noted above), and is not elevated in GFAP-Nrf2 astrocytes (Fig. 1f). Indeed, we observed equal *Jun* induction by tBHQ in Nrf2^–/–^ neurons compared to Nrf2^+/+^ neurons, confirming it as an Nrf2-independent response to tBHQ (Fig. 1i).

**Fig. 2:**
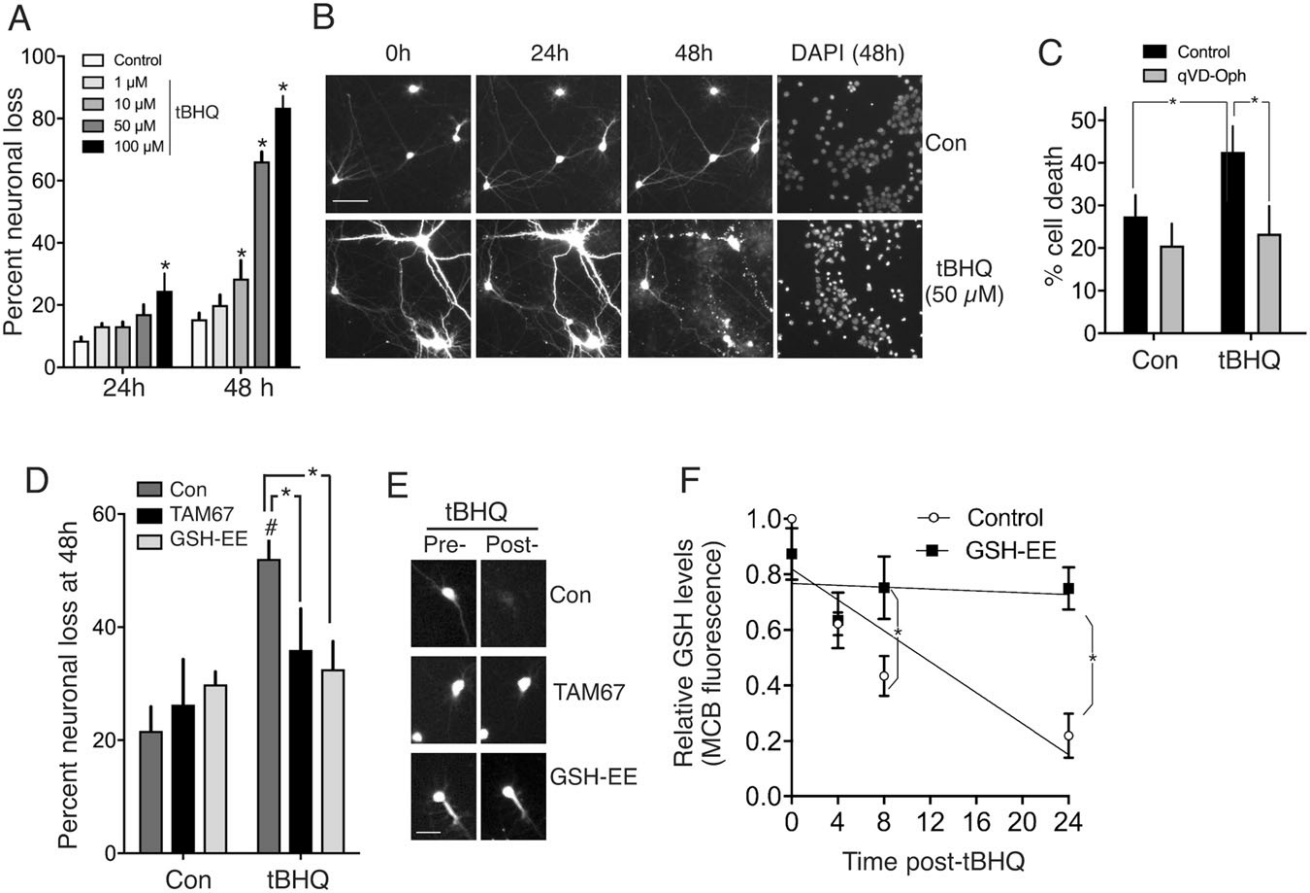
TBHQ promotes Jun-dependent neurotoxicity. **A, B** GFP-transfected cortical neuronal cultures^17^ were imaged at the indicated times post-tBHQ treatment, fixed/DAPI-stained, and viability assessed (death = cell disappearance or neurite fragmentation). *P = 0.004, 0.021, 0.0001, 0.0001; 2-way ANOVA + Dunnett’s post-hoc test, 72-121 cells analysed per condition; *n* = 4 independent biological replicates. **C** Cells were treated ± 50 µM tBHQ ± qVD-Oph (50 µM) and cell death analysed 24 h later. *P = 0.013, 0.0056, 2-way ANOVA + Sidak’s post-hoc test (*n* = 4 independent experiments, 481–582 cells analysed per condition). **D, E** Astrocyte-free neuronal cultures were transfected with eGFP plus either a control vector (ß-globin) or TAM67. The cells were treated with GSH-EE (1 mM) for 1 h where indicated, and then ±50 µM tBHQ. Cell viability was assessed 48 h later. ^#^*P* = 0.0012, 2-way ANOVA + Sidak’s post-hoc test (comparing Control to tBHQ conditions). NB. No significant effect of tBHQ on cell death was observed in TAM67-transfected neurons or GSH-EE treated neurons (relative to corresponding control condition). *P (left-to-right) =0.045 and 0.013, 2-way ANOVA + Sidak’s post-hoc test, n = 6 independent experiments (130-166 cells were analysed per treatment). **F** Neurons were treated with tBHQ (50 µM) for the indicated times and reduced glutathione levels measured using the monochlorobimane method (See^87^ and “Methods”). *P = 0.0436, 0.0003, 2-way ANOVA + Sidak’s post-hoc test (*n* = 4).

*Jun* is induced in neurons by multiple insults, including oxidative/electrophilic stress, and induction can contribute to neuronal apoptosis^29^, so we considered the possibility that *Jun* induction in neurons by tBHQ treatment represents the early signs of a neurotoxic stress response to the electrophilic tBHQ. We observed that concentrations of tBHQ ≥ 10 µM killed neurons in a dose-dependent manner (Fig. 2a,b) which was inhibited by the pan-caspase inhibitor qVD-Oph, suggestive of apoptotic-like cell death (Fig. 2c). Moreover, tBHQ-induced neuronal death was inhibited by TAM67^30^, a dominant negative form of Jun (Fig. 2d,e). The electrophilic/pro-oxidative nature of tBHQ may be a cause of neuronal apoptosis because tBHQ treatment promoted glutathione depletion (Fig. 2f), and both depletion, and neuronal death could be rescued by of cells with a cell permeable form of the (nucleophilic) antioxidant glutathione (Fig. 2d–f), suggesting that oxidative stress is a key factor (though ROS levels were not measured). Thus, not only do cortical neurons fail to mount any Nrf2-dependent transcriptional or proteostasis response to tBHQ, they are vulnerable to neurotoxicity when exposed to tBHQ, triggered via an Nrf2-independent, Jun-dependent mechanism.

### The triterpenoid CDDO^TFEA^ can drive α-synuclein clearance in neurons after epigenetic derepression of Nrf2

**Fig. 3:**
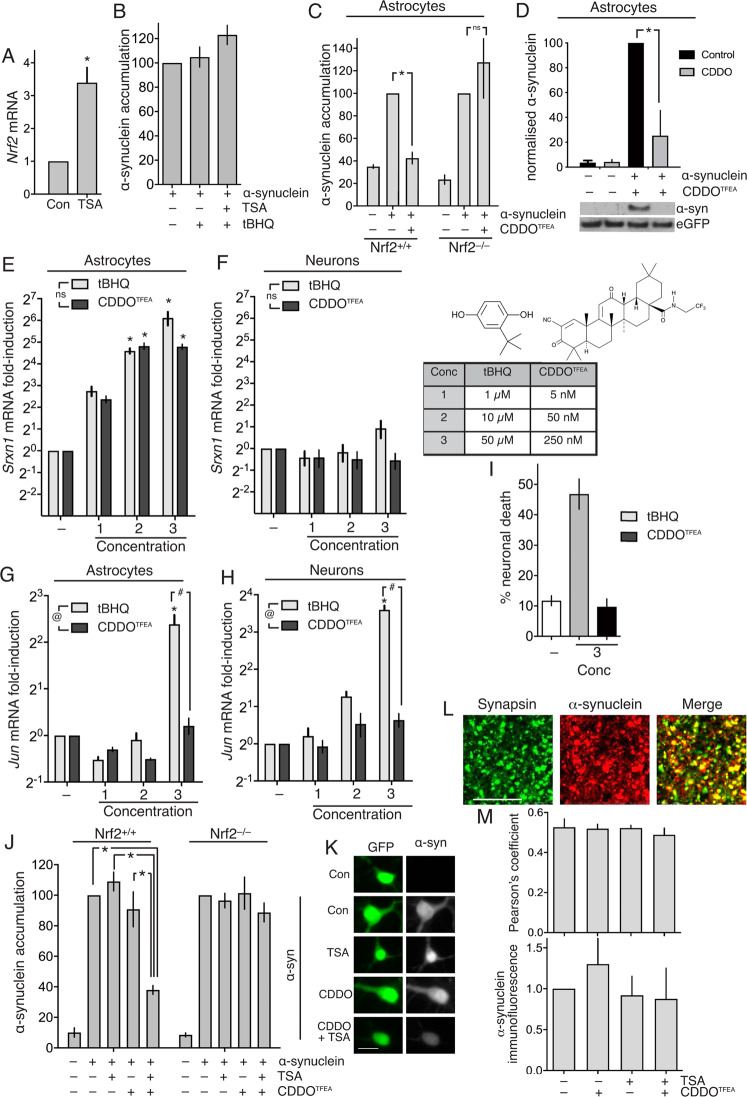
The triterpenoid CDDO^TFEA^ can drive α-synuclein in neurons after epigenetic derepression of Nrf2. **A** Astrocyte-free neuron cultures were treated ± 1 µM TSA for 16 h and *Nrf2* mRNA expression analysed. *P = 0.035 (Student’s *t* test, *n* = 3). **B** Neuronal cultures were transfected on DIV3 with eGFP, plus vectors encoding either α-synuclein or a ß-globin control. 5 days post-transfection, cells were treated ± TSA (1 µM) for 8 h, and subsequently (where indicated) with 10 µM tBHQ for 24 h. Cells were then fixed and processed for α-synuclein immunofluorescence (n = 3-5). **C** Experiment performed as in Fig. 1c except that CDDO^TFEA^ (250 nM) was employed instead of tBHQ. *P = 0.033, 2-way ANOVA + Sidak’s post-hoc test (*n* = 4). **D** Experiment performed as in Fig. 3c except that western blotting was performed to study α-synuclein levels, rather than immunofluorescence, and only WT astrocytes studied. *P = 0.0003, 2-way ANOVA + Sidak’s post-hoc test (*n* = 5). Cortical astrocyte cultures (**E**) or astrocyte-free neuronal cultures (**F**) were treated with different concentrations of tBHQ or CDDO^TFEA^ for 8 h, RNA harvested and expression of the Srxn1 measured by qPCR, normalized to *Rpl13a*. *P values: 0.003, 0.0009, <0.0001, 0.001,0.006, <0.0001; 2-way ANOVA + Dunnett’s post-hoc test (*n* = 5). Concentrations 1, 2, 3 of tBHQ are 1,10, and 50 µM; concentrations 1, 2, 3 of CDDO^TFEA^ are 5, 50, and 250 nM as shown in the table inset in (**F**), as are the structures of tBHQ and CDDO^TFEA^. **G** Same samples as in (**E**), analysed for *Jun* levels. ^@^*P* = 0.0045 (major drug effect, 2-way ANOVA); ^#^*P* < 0.0001 (Sidak’s posthoc test); *P <0.0001, <0.0001, 0.001 (Sidak’s posthoc test, *n* = 5). **H** Same samples as in (**F**), analysed for *Jun* levels. ^@^*P* = 0.0027 (major drug effect, 2-way ANOVA); ^#^*P* < 0.0001 ^(^Sidak’s posthoc test); *P < 0.0001, <0.0001, ^<^0.0001 (Sidak’s posthoc test), (*n* = 5). **I** GFP-transfected astrocyte-free cortical neuronal cultures (DIV10 ^17^) were imaged before and 24 h post-tBHQ (50 µM) or post- CDDO^TFEA^ (250 nM) treatment, fixed/DAPI-stained, and viability assessed. *P = 0.0001, <0.0001, 1-way ANOVA plus Tukey’s post-hoc test, 76–86 cells analysed per condition (*n* = 4). **J, K** Neuronal cultures (WT and Nrf2 KO) were transfected on DIV3 with eGFP, plus vectors encoding either α-synuclein or a ß-globin control. 5 days post-transfection, cells were treated ± TSA (1 µM) for 8 h, and subsequently (where indicated) with 250 nM CDDO^TFEA^ for 24 h. Cells were then fixed and processed for α-synuclein immunofluorescence as for Fig. 1. *P < 0.0001, 1-way ANOVA + Dunnett’s post-hoc test 68–162 cells analysed per condition across *n* = 4 independent experiments. **K** shows example pictures. **L** Example pictures showing the co-localisation of synapsin and endogenous α-synuclein in puncta of a size consistent with being pre-synaptic boutons (scale bar=10 µm). **M** Neurons were treated as indicated, similarly to Fig. 3h, and after 24 h cells were fixed and endogenous synapsin and endogenous α-synuclein analysed by immunofluorescence, and Pearson’s colocalization coefficient calculated (ImageJ JACoP plugin, *n* = 3).

In order to drive endogenous Nrf2-dependent α-synuclein in cortical neurons, it is clear that there is first a need to overcome the lack of Nrf2 mRNA expression. We recently showed that neurons can be rendered more responsive to Nrf2 activators by first de-repressing the Nrf2 promoter by treatment with histone deacetylase (HDAC) inhibitors^17^. Treatment of neurons with the HDAC inhibitor trichostatin A (TSA) rescues histone H3 hypo-acetylation at the Nrf2 promoter and induces Nrf2 mRNA expression^17^ (confirmed in Fig. 3a), which enables them to respond to tBHQ by up-regulating Nrf2 target genes^17^. We followed this protocol in α-synuclein-expressing cortical neurons: pre-treating them with TSA, followed by treatment with tBHQ. However, we observed that even pre-treatment of cortical neurons with TSA failed to enable neurons to mount an α-synuclein clearance in response to tBHQ treatment (Fig. 3b). We hypothesized that the pro-oxidant, electrophilic nature of tBHQ (Fig. 2) makes it sub-optimal as a neuron-focussed Nrf2 activator. This is relevant because oxidative stress can exacerbate α-synuclein aggregation^31,32^ and so the off-target effects of tBHQ may counter-act any on-target effects. We therefore wanted to determine whether alternative Nrf2-activating compounds had a more favourable toxicity profile, or whether all Nrf2-activating compounds, regardless of potency, triggered Nrf2 activation and neurotoxicity at similar concentrations.

We compared tBHQ with another type of Nrf2 activator, the triterpenoid 1[2-Cyano-3,12-dioxool-eana-1,9(11)-dien-28-oyl] trifluoroethylamide (CDDO^TFEA^), also referred to as RTA-404^33^. We chose CDDO^TFEA^ as it is more potent than tBHQ^33^, potentially reducing the scope for off-target effects. We first confirmed that, like tBHQ, CDDO^TFEA^ could promote α-synuclein clearance (by both immunofluorescence and western blot analysis, Fig. 3c,d) and this was not observed in Nrf2-deficient astrocytes (Fig. 3c). We then assessed the dose dependency of both tBHQ and CDDO^TFEA^-induced activation of Nrf2-dependent responses, in comparison to their induction of an Nrf2-independent *Jun* stress response. We performed the comparison on both astrocytes, able to mount Nrf2-dependent and -independent responses, as well as neurons, which only mount an Nrf2-independent stress response. Using induction of the Nrf2 target gene *Srxn1* as a readout, we observed its induction in astrocytes by CDDO^TFEA^ treatment at concentrations around 200 fold lower than tBHQ (Fig. 3e), and as expected, no induction in neurons (Fig. 3f). Importantly however, *Jun* was not comparably induced: while 250 nM CDDO^TFEA^ was equally effective at inducing *Srxn1* in astrocytes as 50 µM tBHQ, it neither induced *Jun* in neurons or astrocytes (Fig. 3g,h), nor did it trigger neurotoxicity (Fig. 3i).

We concluded that CDDO^TFEA^ has a more favourable toxicity profile than tBHQ, so we investigated whether it could induce clearance of over-expressed α-synuclein after derepression of neuronal Nrf2 by TSA pre-treatment. We observed that, while TSA or CDDO^TFEA^ alone failed to promote α-synuclein clearance, a combination of TSA followed by CDDO^TFEA^ successfully achieved this in Nrf2^+/+^, but not Nrf2^–/–^ neurons (Fig. 3j,k), and also induced classical Nrf2 target genes Hmox1 and Nqo1 (Fig. S2a,b). Thus, epigenetic de-repression of Nrf2 in cortical neurons enables Nrf2-dependent proteostasis processes to be pharmacologically induced by CDDO^TFEA^, a non-stress inducing Nrf2 activator. We next wanted to determine the influence of this intervention on endogenous α-synuclein, which is primarily pre-synaptic in localisation. We therefore repeated the conditions used in Fig. 3j, but studied endogenous α-synuclein, and its pre-synaptic localisation, using synapsin as a presynaptic marker. Detection of endogenous α-synuclein required longer exposure time than detection of over-expressed α-synuclein, but its synaptic co-localisation with synapsin was apparent (Fig. 3l), its co-localisation with synapsin was unaffected by TSA or CDDO^TFEA^ alone or in combination (Fig. 3m, upper), and overall fluorescence intensity unaffected (Fig. 3m, lower), supporting the concept that this intervention preferentially clears inappropriately accumulated α-synuclein, rather than endogenous synaptic α-synuclein.

**Fig. 4:**
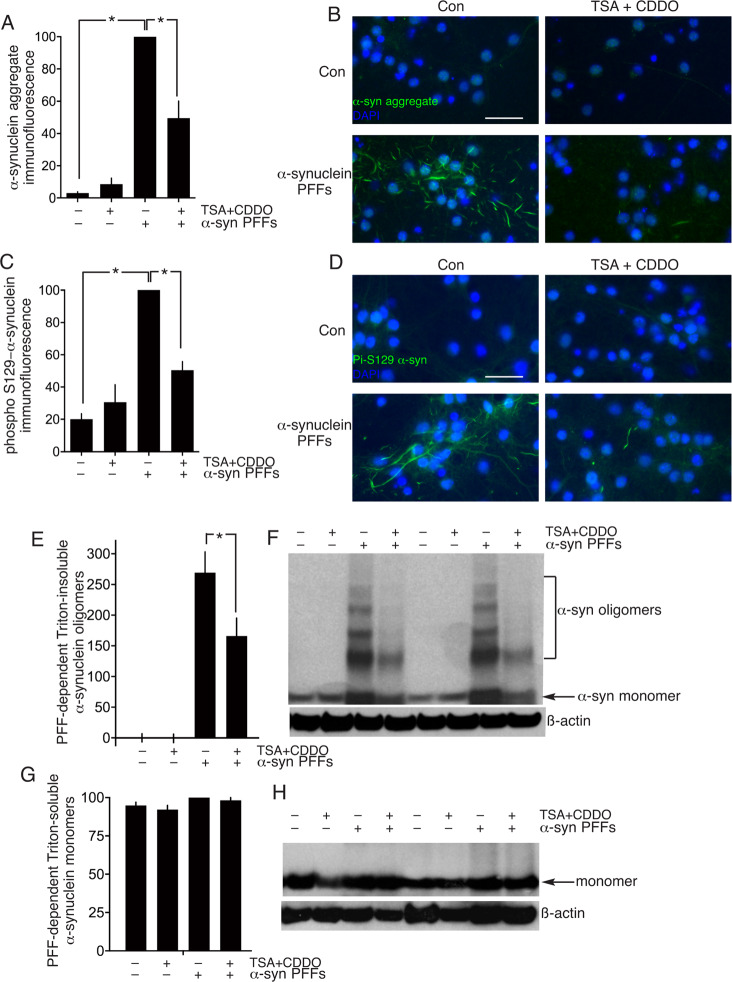
CDDO^TFEA^ and HDAC inhibition promote clearance of α-synuclein aggregates. **A–D** Neuronal cultures were exposed where indicated to α-synuclein pre-formed fibrils (PFFs) on DIV4 until DIV15 to cause α-synuclein aggregate formation. On DIV15 the neurons were treated where indicated with TSA (1 µM, 8 h) followed by CDDO^TFEA^ (250 nM, 24 h). Neurons were fixed and processed for immunofluorescence using an α-synuclein aggregate conformation-specific antibody (**A**, **B**) or a phospho-(Ser-129)- α-synuclein specific antibody (**C**, **D**) A: *P = 1.5E–09, 9.5.E–06, 1-way ANOVA + Sidak’s post-hoc test (*n* = 5); C: *P = 1.1E–06, 1.2E–04, 1-way ANOVA + Sidak’s post-hoc test (*n* = 4). **B** and **D** show example pictures relating to (**A**) and (**C**) respectively. Scale bar: 50 µm. **E, F** Neuronal cultures were treated as per (**A**–**D**), Triton-insoluble proteins were extracted (see “Methods”) and western analysis of oligomeric α-synuclein performed, normalized to ß-actin. *P = 0.0035, 1-way ANOVA + Sidak’s post-hoc test (*n* = 8). **G**, **H** Neuronal cultures were treated as per (**E**), except that Triton-soluble proteins were extracted (see “Methods”) and α-synuclein monomer expression quantified normalized to ß-actin (*n* = 8).

As an alternative to studying the capacity of TSA + CDDO^TFEA^ to clear over-expressed α-synuclein over-expression, we used a system of exposing α-synuclein pre-formed fibrils (PFFs) to neurons for 9 days to promote α-synuclein aggregation. We first studied α-synuclein aggregate presence using an aggregate conformation-specific antibody by immunofluorescence and observed that TSA + CDDO^TFEA^ caused a reduction of fibril presence by ~50% (Fig. 4a,b). This reduction is mirrored when analysing phosphorylation of alpha-synuclein on serine 129, and event that occurs preferentially on alpha-synuclein aggregates (Fig. 4c,d). We also performed western blot analysis of triton-insoluble, oligomeric alpha-synuclein in PFF-exposed neurons, and also observed a reduction by TSA + CDDO^TFEA^ treatment (Fig. 4e,f).

We also analysed levels of endogenous alpha-synuclein by western blot. Endogenous alpha-synuclein is extractable by Triton detergent and runs as a monomer on a western blot. TSA + CDDO^TFEA^ treatment had no influence on Triton-soluble alpha-synuclein monomer levels (Fig. 4g,h). Also of note, exposure of neurons to PFFs also had no effect on Triton-soluble alpha-synuclein monomer levels, consistent with their Triton-insoluble nature (Fig. 4g,h). Collectively these data do not support the hypothesis that endogenous alpha-synuclein is depleted by TSA + CDDO^TFEA^ over the course of the experiment, but we acknowledge that studying later timepoints in the future may be desirable.

### dCas9-based transcriptional activation complexes targeted to the Nrf2 promoter induce α-synuclein clearance

**Fig. 5:**
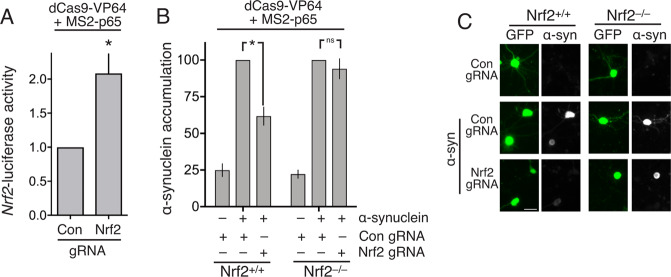
dCas9-based transcriptional activation complexes targeted to the Nrf2 promoter induce α-synuclein clearance. **A** Neurons were transfected at DiV 8 with dCas9-VP64, p65 and sgRNAs targeting the Nrf2 promoter (controls were empty sgRNA vectors) with Nrf2-promoter Firefly luciferase and TK-Renilla control. Luciferase activity was measured at 96 h. *P = 0.032, Student’s *t* test (*n* = 4). **B** Nrf2^+/+^ and Nrf2^−/−^ cortical neurons were transfected as in (**A**) in addition to α-synuclein expression vector and eGFP transfection marker. At Div 11 cells were fixed and processed for GFP and α-synuclein immunofluorescence as for Fig. 1. *P = 0.001, 2-Way ANOVA + Bonferroni’s multiple comparison test, 192–573 cells analysed per condition (*n* = 7 WT and 5 Nrf2 KO). **C** Example pictures.

Finally, we investigated whether a more targeted activation of the neuronal Nrf2 promoter could induce α-synuclein clearance. We employed a synthetic transcription factor complex based on nuclease-defective Cas9 fused to the viral transactivation domain VP64, in combination with three specifically designed sgRNAs to target the complex to the Nrf2 promoter^34^. In addition, the sgRNAs contained a minimal hairpin aptamer, appended to both the sgRNA tetraloop and stem loop 2, which binds to dimerized bacteriophage MS2 coat proteins (hereafter, sgRNA(MS2)). Co-expression of a fusion protein comprised of MS2 and the p65 trans-activating subunit of NF-kB, leads to its recruitment to the dCas9-VP64/sgRNA complex, and further strengthens the transactivating power of the complex^34^. We confirmed that co-expressed dCas9-VP64 and MS2-p65 could activate an Nrf2 promoter-driven luciferase reporter, when co-expressed with Nrf2 promoter targeting sgRNA(MS2), but not with control sgRNA(MS2) (Fig. 5a). Furthermore, we found that this synthetic Nrf2-activating dCas9-transactivator complex could prevent the accumulation of α-synuclein (Fig. 5b,c). To determine whether the prevention of the accumulation of α-synuclein by the Nrf2-activating dCas9-transactivator complex was due to any off-target effects, we repeated the experiment in cortical neurons prepared from Nrf2^-/-^ mice. We observed no effect of the synthetic Nrf2-activating dCas9-transactivator complex on α-synuclein accumulation in Nrf2^−/−^ neurons, confirmation of the Nrf2-dependency of the process (Fig. 5b,c). Thus, the targeted activation of the neuronal Nrf2 promoter using a synthetic dCas9-transactivator complex is sufficient to induce an Nrf2-dependent proteostasis response.

## Discussion

Research into the protective effects of Nrf2 activation in the CNS has hitherto mainly focussed on its activation in astrocytes. Activation of astrocytic Nrf2 can promote neuroprotection via a non-cell-autonomous mechanism due to enhanced production and release of glutathione, which is then utilized by nearby neurons to enhance their own antioxidant defences^4,35–38^. Nrf2 activators can also repress brain inflammatory deregulation through modulating macro- and micro-glial responses, mechanisms thought to be at the heart of Multiple Sclerosis drug Tecfidera’s (dimethyl fumarate) mechanism of action^39,40^, although Nrf2-independent actions of Tecfidera have also been reported^41,42^. However, while the activation of Nrf2 in glia can promote neuroprotection indirectly via inhibition of inflammatory or redox dysregulation in the brain, a significant proportion of Nrf2-dependent genes can only exert their cytoprotective effects cell-autonomously. The influence of many Nrf2 regulated detoxification enzymes, cellular oxidoreductases, molecular chaperones, proteostasis machinery and other response genes are likely to only be cytoprotective in the cell in which they are expressed^43^. As such, the inability of cortical neurons to express sufficient Nrf2 to support a response to Nrf2 activators (Fig. 1h, Supplemental Table S2, 17) suggests that they lack an important adaptive homoeostatic pathway. The biological reason for the repression of Nrf2 expression in cortical neurons early in development appears to be to facilitate their maturation by providing a more flexible redox environment to potentiate key signalling pathways^17^. However, the resultant lack of Nrf2 into maturity potentially renders neurons vulnerable to particular insults, and reliant on support from surrounding glia, which exhibit protective Nrf2 activation following mild trauma such as preconditioning episodes of ischaemia^44,45^, unlike neurons which have distinct transcriptional and post-transcriptional responses to ischaemia^46^. The findings that artificial Nrf2 over-expression in neurons can protect them against diverse insults, including oxidative and ethanolic stress, amyloid-induced deficits, and nerve crush-induced injury^47–50^ as well as α-synuclein clearance^18^ certainly suggests that Nrf2 activation in neurons may be beneficial. However, this must be viewed in the context of the challenges associated with activating an Nrf2 response in cells where Nrf2 levels are so low, due to transcriptional repression, that Nrf2 target genes are not activated by tBHQ (Fig. 1h). Of note, it is apparent that some Nrf2 target genes can be induced in neurons by synaptic activity by Nrf2-independent routes, contributing to the general protective effects of neuronal activity^51–55^. However, the complement of Nrf2 target genes induced in this way is far from comprehensive, and insufficient to fully compensate for Nrf2 hypofunction. Another question is whether de-repression of Nrf2 in mature neurons is safe, or whether continued Nrf2 repression is needed. Of note, viral over-expression of Nrf2 in neurons of the hippocampus ameliorates deficits in an Alzheimer’s disease model^48^, suggesting that Nrf2 de-repression could be well-tolerated.

Our studies suggest that, while Nrf2 activators alone cannot induce Nrf2-target gene expression or α-synuclein clearance in cortical neurons, epigenetic de-repression of Nrf2 prior to Nrf2 activator treatment can achieve this (though it would be desirable in the future to study other neuronal types affected by α-synuclein aggregates such as midbrain dopaminergic neurons). Since α-synuclein aggregates can be degraded by both autophagic clearance and proteosomal degradation, and their formation can be prevented in the first place by molecular chaperones and the UPR^56^, several mechanisms may contribute to α-synuclein clearance following Nrf2. For example, Nrf2 antagonizes UPR-associated cytotoxicity and associated CHOP10 expression^7^. Analogous results have been obtained in mouse embryonic fibroblasts, where targeted deletion of Nrf2 sensitizes them to tunicamycin-induced cytotoxicity^8^. Nrf2-driven gene expression can repress ER stress and UPR through the control of glutathione biosynthesis and recycling enzyme genes, essential for maintaining disulphide chemistry in the ER^9^. In another study, Nrf2 target gene Gpx8, a KDEL-motif containing ER-localised glutathione peroxidase, was seen to repress UPR activation. Keeping an appropriate redox balance is of direct relevance to α-synuclein aggregation, since oxidizing conditions promote α-synuclein aggregation^57,58^, potentially via oxidative or nitrative modification of α-synuclein^59,60^. In addition to modulating the UPR, Nrf2 can control other key arms of cellular proteostasis machinery, the UPS and also autophagic protein clearance. Nrf2 activators promote UPS activity through the regulation of multiple proteasome subunit genes in liver cells, fibroblasts and human ES cells, including Psma and Psmb genes^61,62^, Similarly, Nrf2 can modulate macroautophagy in part by regulating expression of cargo recognition gene p62/SQSTM1, as well as genes involved in autophagy initiation, autophagosome formation, elongation and clearance^10,11^. Of course, Nrf2 derepression via HDAC inhibition may not be feasible as a therapeutic strategy given the non-specific nature of this intervention. On the other hand that Valproate, which inhibits Class I/II HDACs (among other things) like TSA, is a tolerated drug prescribed for certain CNS disorders (epilepsy, bipolar disorder, migraine) and so may achieve Nrf2 de-repression without damaging side effects. This warrants further investigation.

Our study has also highlighted the importance of the Nrf2 activating strategy employed. The Nrf2 promoter must be first de-repressed, such as with a histone deacetylase inhibitor, prior to an Nrf2 activator being employed (Fig. 3a). The classical Nrf2 activator tBHQ, while effective in driving Nrf2-dependent gene expression, caused off-target effects in neurons, promoting glutathione depletion and oxidative neurotoxicity associated with Nrf2-independent *Jun* induction. The electrophilic nature of tBHQ, important for modifying redox-sensitive cysteine residues of Keap1 in order to activate Nrf2, is also likely responsible for the neurotoxicity, since it was repressed by nucleophilic antioxidant glutathione (Fig. 2d,e). Jun transcription is known to be regulated by JNK, whose upstream activators include redox sensitive signalling molecules such as ASK1^63^.

Neuronal vulnerability to tBHQ contrasts with other cell types, and may be due to their relatively weak intrinsic antioxidant defences, itself a consequence of Nrf2 hypofunction^17^. However, the relationship between on- and off-target effects of Nrf2 activators appears to be compound-specific, since we found that CDDO^TFEA^ could activate Nrf2 at concentrations that did not induce a stress response in neurons. Their distinct molecular structure and chemistry of these two molecules must underlie their differences. Electrophilic compounds of different structures may have different relative affinities for electron donor groups. It is possible that CDDO^TFEA^ preferentially reacts with key Keap1 cysteine residues compared to other “off-target” nucleophiles whose oxidation leads to non-specific oxidative stress and Jun activation. However in the absence of any guiding principles to explain these differences, Nrf2 activators should be assessed for their on- vs. off-target effects, and no electrophilic Nrf2 activator is likely to be completely devoid of off-target effects. CDDO^TFEA^, also referred to as RTA-404, is structurally highly related to another synthetic triterpenoid RTA-408 (trade name omaveloxolone), both of which are licensed to Reata Pharmaceuticals. RTA-408 (see Fig. 3) has extensive human and primate safety and pharmacokinetic data, both when taken orally and topically applied^64–67^ and is in clinical trials for Friedreich ataxia and mitochondrial myopathy^68^, with positive Phase 2 data recently reported for Friedreich ataxia^69^. In contrast, tBHQ is a food additive used as a preservative (E319) though levels are strictly limited and several studies point to potential carcinogenic or cytotoxic effects, despite its efficacy as an Nrf2 activator^70–73^.

Other strategies for reducing systemic side effects of electrophilic Nrf2 activators are also being developed, such as the use of pro-drugs such as carnosic acid, whose electrophilic nature is only exposed upon oxidative modification, and thus are only active in cells experiencing oxidative stress, and have been found under certain circumstances to induce Nrf2 activity in neurons^74^

As an alternative to the use of small molecules to first de-repress, then activate Nrf2, we show that targeting of a dCas9-based transcriptional activation complex to the Nrf2 promoter is sufficient to drive Nrf2-dependent α-synuclein clearance (Fig. 5). Two very recent studies have shown the potential for dCas9-based targeted gene activation to alter neuronal phenotype. Fragile X Syndrome neurons have been rescued through targeted demethylation of the *FMR1* gene^75^, and neuronal differentiation has been promoted in stem cells through targeted, inducible activation of *NEUROG2*^76^. Ours represents a third example of what is likely to represent a useful and flexible approach to manipulate neuronal properties via gene activation. A theoretical advantage of the dCas9-based systems is their selectivity and lack of off-target effects^77^, and our demonstration that Nrf2-promoter targeted complexes drive α-synuclein clearance in Nrf2^+/+^, but not Nrf2^−/−^ neurons represents strong evidence that they are acting specifically via Nrf2 induction. As gene therapy technologies develop, these or similar approaches may become a viable therapeutic option for controlling Nrf2 activity in neurons. As a master regulator of antioxidant, detoxification, and proteostasis genes, the effective regulation of Nrf2-driven gene expression has the potential to antagonize multiple pathways driving neurodegeneration.

## Materials and methods

### Cell culture

Astrocytes and neurons were cultured from mixed sex E17.5 Nrf2^+/+^ or Nrf2^−/−^ mice (on a C57BL/6 J background) as described^45,78,79^. Nrf2^−/−^ mice have been previously described^80^ and were obtained from Jackson Laboratories (stock no. 017009). Cortices were dissected in dissociation medium containing 1 mM kynurenic acid. Dissociation medium consists of: 81.8 mM Na_2_SO_4_, 30 mM K_2_SO_4_, 5.84 MgCl_2_, 252 µM CaCl_2_, 1 mM HEPES, 20 mM Glucose and 0.002% Phenol Red; 10x kynurenic acid solution contains: 10 mM kynerunic acid, 100 mM MgCl_2_, 5 mM HEPES and 0.002% Phenol Red. Once dissected, cortices were enzymatically digested with papain (Sigma) mechanically dissociated, and plated on poly-D-Lysine and Laminin (Sigma) coated 24-well plates. Neurons were maintained in Neurobasal-A medium containing B-27 (Life Technologies) and 1% Rat Serum, and treated with 4.8 µM of the anti-mitotic cytosine-arabinoside (AraC) at DIV 4. The addition of AraC at this time point ensures a robust cell culture of 90% NeuN-positive neurons and 10% GFAP-positive astrocytes;^45^ for pure astrocyte-free neuronal cultures, AraC was added at DIV 0, limiting the number of GFAP-positive astrocytes to <0.2%^17,51^. Astrocytic cultures were maintained in DMEM with 10% Foetal Bovine Serum (Life Technologies) for 7 days, dissociated with Trypsin and sub-cultured once. For stimulations, cells were transferred to a trophically deprived medium (TMo) containing 10% MEM, 1% Anti-Anti and 89% Salt/Glucose/Glycine medium consisting of: 114 mM NaCl, 0.219% NaHCO_3_, 1 mM MgCl_2_, 2 mM CaCl_2_, 10 mM HEPES, 1 mM glycine, 30 mM glucose, 0.5 mM sodium pyruvate and 0.002% Phenol Red.

### Transfections

For neuronal death assays DIV 7 cells were transferred to TMo supplemented with 1% Insulin Transferrin Selenium solution (Life Technologies) and transfected using 2.33 µl per well Lipofectamine 2000 (ThermoFisher). The neuronal cultures were then transfected with 0.15 µg pEF-GFP and 0.4 µg Globin or TAM67; maintained in TMo for 48 h and treated 24 h with tBHQ (Sigma) reduced Glutathione ethyl-ester (GSHee, Sigma) or CDDO-trifluoroethylamide^33^ (CDDO-^TFEA^, a gift from Professor Michael Sporn). For α-synuclein experiments neurons were transfected with 0.15 µg pEF-GFP, 0.3 µg of human α-Synuclein (a gift from Dr Tilo Kunath) or Globin; then treated with Trichostatin A (Tocris) for 8 h before 24 h stimulation with tBHQ or CDDO^TFEA^. For analysis of α-synuclein levels in Nrf2 wildtype/knockout cells, cultures were transfected at DIV 4 to facilitate ectopic expression in both neurons and astrocytes in the same well. For dCas9-Nrf2 experiments, sgRNA targeting the mouse Nrf2 promoter were designed using the Feng Zhang lab online resource http://crispr.mit.edu with the following sequences cloned into the sgRNA(MS2) backbone^34^ (Addgene): sg-146bp: ACA AGA CGG GGG CCA GTG GA, sg-360bp: GCT GCT AAT CTC TAG CAA GG, sg-452bp: CCC GGC CCG TGC GCT GCT AT; number indicates number of nucleotides from beginning of exon 1 of Nfe2l2. Neurons were transfected at DIV 7 with 0.1 µg pEF-GFP, 0.25 µg α-synuclein or globin, 0.15 µg of dCas9-VP64 fusion construct, 0.15 µg p65, and either 0.05 µg of each Nrf2-targetting sgRNA or 0.15 µg of empty sgRNA(MS2) vector. Neurons were maintained for 4 days then fixed and processed for imaging. For ARE-luciferase assays, neurons were transfected with 0.2 µg of ARE-luciferase, 0.15 µg of Tk-Renilla and 0.3 µg of TAM67 or Globin. After 24 h neurons were stimulated with tBHQ for 24 h. For Nrf2-luciferase assays, complementary forward and reverse oligos (Sigma) of the following sequence of the mouse Nrf2 promoter were annealed and cloned into pGL3.10 (Promega):TAG GCC TTT GCG GGG GGC CCT CGG GTC CTT GCC CTG CCC CTG TAC GCG ATT CCA AGC TCT TGC CCC GCC CCT TAC CCC GCC TCC ATG CCC TTGA. Neurons were transfected with 0.15 µg of dCas9-VP64 fusion construct, 0.15 µg p65, 0.2 µg Nrf2-luciferase, 0.1 µg Tk-Renilla and either 0.05 µg of each Nrf2-targetting sgRNA or 0.15 µg of empty sgRNA(MS2) vector, and were maintained for 4 days. Luciferase assays were performed using the Dual Glo Kit (Promega) using a FLUOStar OPTIMA (BMG Labtech) with Firefly luciferase-reporter gene activity normalised to Renilla control.

### Immunohistochemistry and imaging

All imaging was performed using a Leica AF6000 LX system and DFC350 FX digital camera. For α-synuclein assays, transfected cells were stimulated as indicated, then immunofluorescence performed essentially as described^81^. Briefly, cells were fixed with 4% paraformaldehyde for 20 min at room temperature (21 °C), washed with PBS, then cell membranes were permeabilised by 0.5% NP40 (Life Technologies) treatment for 5 min. Neurons were subsequently incubated with mouse anti α-synuclein (BD Biosciences) at 1:1000 dilution for 3 h at room temperature, followed by Cy3-conjugated donkey anti mouse secondary antibody (1:250 dilution, 2 h at room temperature; Jackson ImmunoResearch) and FITC-conjugated goat anti GFP antibody (1:500 dilution, 2 h at room temperature; Abcam). A glass coverslip was then mounted using DAPI-containing Vectashield (Vector Labs). For any single experiment, exposure time is constant for all pictures and conditions, and is set low enough such that no pixel in any cell is saturated. We performed an experiment whereby we altered the amount of α-synuclein plasmid in our transfection mixture, keeping the amount of eGFP co-transfection plasmid constant, and measured the level of α-synuclein expression by immunofluorescence. This revealed an approximately linear relationship between the quantity of α-synuclein plasmid, and the level of α-synuclein expression detected (Fig. S1a, b). Images were chosen for analysis based on GFP-signal not α-synuclein, and were analysed with the experimenter blind to condition using ImageJ software, measuring integrated density of α-synuclein immuno-fluorescence of cell soma. For these experiments we use morphology to distinguish astrocytes from neurons, but used neuronal and astrocytic markers to validate this as an approach. Briefly, mixed neuron-astrocyte cultures were transfected with eGFP and co-stained with either GFAP (astrocytes) or NeuN (neurons). Then, blind to the GFAP or NeuN staining, pictures of eGFP-expressing cells were classified as neurons or astrocytes by 1^st^ author Paul Baxter, based on morphology, after which an independent person determined which of these cells were GFAP or NeuN-positive. Of 80 cells classified as astrocytes based on morphology, 98.7% were GFAP-positive and 1.3% were NeuN-positive. Of 134 cells classified as neurons based on morphology, none were GFAP-positive and 100% were NeuN-positive. Thus, morphology is an accurate way of distinguishing neurons from astrocytes, as long as the experimenter is experienced, and the approach is validated. The following approximate number of cells were analysed: Nrf2 WT/KO astrocytes (22 astrocytes per condition across 4 independent experiments), Nrf2 WT/KO neurons (65 neurons per condition across 4 independent experiments), Trichostatin treated wildtype neurons (102 WT neurons and 93 Nrf2^–/–^ neurons per condition across 4 independent experiments) dCas9-VP64 transfected neurons (344 wildtype neurons and 209 Nrf2 KO neurons per condition across 7 and 5 independent experiments).

For neuronal death assays, GFP-transfected neurons were imaged and their locations mapped using the Leica “Mark and Find” software application. Neurons were allowed 3 h to re-equilibrate and then stimulated as indicated. Images were taken of saved locations at 24 and 48 h post stimulation, with at least two wells per condition and two pictures per well. Cell death was assessed by counting the number of surviving GFP-positive neurons pre and post-stimulation, with the user blind to the image analysis. Neuronal death was easily identifiable by the replacement of a healthy GFP-expressing cell with the presence of fragmented neurites and fluorescent cell debris. In addition, cell viability was measured using the Cell Titre-Glo assay kit (Promega).

For measurement of α-synuclein and synapsin colocalization, untransfected neurons were treated as above with Trichostatin or CDDO^TFEA^, then fixed, permeabilised and incubated over-night at 4oC with mouse α-synuclein antibody and rabbit synapsin antibody (1:1000 dilution; Synaptic Systems), followed by 2 h incubation with Alexa Fluor 594 conjugated anti-mouse and Alexa Fluor 488 conjugated anti-rabbit antibodies. Images were taken at 60x magnification using a Nikon A1R confocal microscope. Pearson’s colocalization coefficient was calculated using the JACop plugin for ImageJ^82^, with 18 images taken per condition across 3 independent experiments.

Glutathione depletion assays were performed as described (Baxter et al., 2015), substituting tBHQ (50 µM) for H_2_O_2_. Briefly, cells were treated with GSH-ee 1 mM for 1 h, then with tBHQ 50 µM for the times indicated. thirty minutes before the end of stimulation, neurons were treated with 50 μM MCB, and allowed to incubate at 37 °C. Cells were then washed once with fresh TMo, and lysed in K_2_HPO_4_ buffer containing 0.5% Triton-X-100. Lysates were centrifuged at 15,700 × *g* (13,000 r.p.m.) at 4 °C for 10 min, and supernatants were transferred to a black 96-well plate for fluorescence measurement (excitation 405 nm, emission 520 nm) with a FLUOstar OPTIMA. Lysates were then assayed for protein concentration using a BCA assay, to which fluorescence values were normalized to.

### RNA Isolation, qPCR and RNA-sequencing

RNA was isolated using the High Pure RNA Isolation Kit (Roche) according to the manufacturer’s instructions, including a column based 15 min DNAse I treatment. For qPCR, cDNA was synthesised from 1 to 3 µg of RNA using the Transcriptor First Strand cDNA Synthesis kit (Roche) using a mix of both random Hexamer primers and anchored Oligo(dT)_18_ primers. qPCRs were run on a Stratagene Mx3000P qPCR system (Agilent Technologies) using FastStart SYBR Green Master (Rox) mix (Roche) using 6 ng of initial RNA per 15 µl qPCR reaction and 200 nM of forward and reverse primers to standard PCR and amplification conditions. Gene of interest expression was normalised to RPL13A mRNA expression. The following primers were used:

*Rpl13a* - F: GATGAATACCAACCCCTCC, R: CGAACAACCTTGAGAGCAG.

*Srxn1* – F: TCTCAAAGGTCAGTTCAGGAG, R: TTTGCTCGAATGTGTTTGTC.

*Jun* – F: GGAGAGCCGCTGTTGCTGGGA, R: TCCGCTAGCACTCACGTTGGTA.

*Nrf2* (*Nfe2l2*) – F: CAGCTCAAGGGCACAGTGC, R: GTGGCCCAAGTCTTGCTCC.

*Slc7a11* (*xCT*) – F: ATACTCCAGAACACGGGCAG, R: AGTTCCACCCAGACTCGAAC

*Hmox1* – F: AGCACAGGGTGACAGAAGAG, R: GGAGCGGTGTCTGGGATG

For RNA-sequencing, libraries were prepared from RNA samples by Edinburgh Genomics using the Illumina TruSeq stranded mRNA-seq kit, according to the manufacturer’s instructions (Illumina). The libraries were pooled in equimolar proportions and sequenced on an Illumina HiSeq 2500 platform in high output mode (v4 chemistry). Sequencing was performed to a depth of ~35 million paired-end reads per sample, with 3 biological replicates per condition. Sequencing reads were mapped to the *Mus Musculus* (mm10) reference genome using the STAR RNA-seq aligner version 2.5.3a^83^, with a summary of per-gene read counts generated from the mapped reads with featureCounts version 1.5.2^84^ using annotations from Ensembl version 90^85^. Relative expression levels of genes are expressed as fragments per million reads per kilobase of message (FPKM). Differential expression analysis was performed using DESeq2 (R package version 1.16.1)^86^ with a significance threshold set at a Benjamini-Hochberg-adjusted p-value <0.05. Raw data are deposited at EBI (E-MTAB-5688).

### Exposure to neurons of α-synuclein pre-formed fibrils

Mouse neurons were cultured as described, with AraC added to feeding medium at Div 0, except as follows. At Div 4, cell media was collected, and recombinant 1 µg/ml human α-Synuclein protein aggregate (Abcam; ab218819) was added, vortexed and replaced on cells. Neurons were maintained till DiV 15 by the replacement of 50% media with Neurobasal-A + B27 every 3 days. Cells were then moved into TMo, stimulated for 8 h with 1 µM Trichostatin A, then 24 h with 250 nM CDDO-TFEA. Neurons were then either processed for western blot as described below or fixed, permeabilised and probed with fibril-conformation specific anti- α-Synuclein antibody (Abcam 1:5000, MJFR-14-6-4-2 ab209538) or anti phospho-Serine 129 α-Synuclein (Abcam, 1:500; MJF-R13(8-8), ab168381); images were analysed using ImageJ, with DAPI images used to mask nuclear stain to analyse specific neurite stain only.

### Western blotting

For western blotting two methods of cell isolation were utilised. Monocultured astrocytes were isolated using RIPA buffer (10 mM Tris pH 7.4, 100 mM NaCl, 1 mM EDTA, 1 mM EGTA, 1% Triton X, 10% glycerol, 0.1% SDS, 0.5% deoxycholate) containing 1:100 complete Mini protease inhibitor cocktail (Roche), then diluted to 1 µg/µl with the addition of 1:10 NuPage Sample Reducing Agent and 4x NuPage LDS Sample buffer (Invitrogen). For PFF-treated samples, neurons were initially isolated in Triton Isolation buffer (100 mM NaCl, 20 mM HEPES, 1% Triton-X, pH 7.4), centrifuged (12000 × *g*, 20 min, 4 °C), supernatants were collected and pellets lysed in Triton Insoluble/SDS Isolation buffer (150 µM NaCl, 20 mM HEPES, 1% Triton-X, 1% sodium deoxycholate, 1% SDS, 1 mM DTT, pH 7.4) overnight at 4 °C with constant rotation. Samples were centrifuged as before, supernatants were again collected, PhosSTOP phosphatase inhibitor and complete Mini protease inhibitor cocktail (1:100) were added to all Isolation buffers before use; samples were then diluted to 1 µg/µl in reducing agent and LDS sample buffer as above. Prior to running, samples were boiled at 100 °C for 4 min; then 10 µg protein was loaded to precast NuPage Bis-Tris 4-12% gradient gels (Invitrogen) and subjected to electrophoresis in MES buffer. Western blotting on to nitrocellulose membrane was then performed using the Xcell Surelock system (Invitrogen) as per manufacturer’s instructions. Following protein transfer, membranes were blocked for 1 h at room temperature with 5% (w/v) non-fat dried milk in TBS with 0.1% Tween 20. The membranes were incubated at 4 °C overnight with the primary antibodies diluted in blocking solution: anti-α-Synuclein (1:1000, BD Transduction Laboratories), anti-β-Actin (1:2000, Abcam). Western blots were visualised using mouse HRP-linked secondary antibody (1:1000, Thermo) followed by chemiluminescent detection on Kodak-X-Omat film. Films were scanned and densitometric analysis preformed using ImageJ, with all values normalised to β-Actin loading control.

### Statistical analysis

Statistical testing of the RNA-seq data is described in the RNA-seq methods section. Sample size for experiments was based on powering an experiment at 80% to detect a 30% effect size, based on the variance of data in previously published experiments from our laboratory. Other testing involved a 2-tailed paired Student’s t-test, or a one- or two-way ANOVA followed by Sidak’s or Dunnett’s post-hoc test, as indicated in the legends. For t-tests, variance was generally found to be similar, abrogating the need for Welsh’s Correction. No data were exlcuded. Throughout the manuscript, independent biological replicates are defined as independently performed experiments on material derived from different animals.

## Supplementary information

Supplemental Figures and Legends

Supplemental Tables 1 and 2

## References

[1] Tebay LE (2015). Mechanisms of activation of the transcription factor Nrf2 by redox stressors, nutrient cues, and energy status and the pathways through which it attenuates degenerative disease. Free Radic. Biol. Med.

[2] Kobayashi EH (2016). Nrf2 suppresses macrophage inflammatory response by blocking proinflammatory cytokine transcription. Nat. Commun..

[3] Taguchi K, Yamamoto M (2017). The KEAP1-NRF2 system in cancer. Front Oncol..

[4] Johnson DA, Johnson JA (2015). Nrf2–a therapeutic target for the treatment of neurodegenerative diseases. Free Radic. Biol. Med..

[5] Kobayashi A (2006). Oxidative and electrophilic stresses activate Nrf2 through inhibition of ubiquitination activity of Keap1. Mol. Cell Biol..

[6] Satoh T, McKercher SR, Lipton SA (2013). Nrf2/ARE-mediated antioxidant actions of pro-electrophilic drugs. Free Radic. Biol. Med..

[7] Lee S (2012). Involvement of the Nrf2-proteasome pathway in the endoplasmic reticulum stress response in pancreatic beta-cells. Toxicol. Appl. Pharm..

[8] Cullinan SB (2003). Nrf2 is a direct PERK substrate and effector of PERK-dependent cell survival. Mol. Cell Biol..

[9] Zanotto-Filho A (2016). Alkylating agent-induced NRF2 blocks endoplasmic reticulum stress-mediated apoptosis via control of glutathione pools and protein thiol homeostasis. Mol. Cancer Ther..

[10] Jain A (2010). p62/SQSTM1 is a target gene for transcription factor NRF2 and creates a positive feedback loop by inducing antioxidant response element-driven gene transcription. J. Biol. Chem..

[11] Pajares M (2016). Transcription factor NFE2L2/NRF2 is a regulator of macroautophagy genes. Autophagy.

[12] Walden H, Muqit MM (2017). Ubiquitin and Parkinson’s disease through the looking glass of genetics. Biochem J..

[13] Hipp MS, Kasturi P, Hartl FU (2019). The proteostasis network and its decline in ageing. Nat. Rev. Mol. Cell Biol..

[14] Newton TM, Duce JA, Bayle ED (2019). The proteostasis network provides targets for neurodegeneration. Br. J. Pharmacol..

[15] Shih AY (2003). Coordinate regulation of glutathione biosynthesis and release by Nrf2-expressing glia potently protects neurons from oxidative stress. J. Neurosci..

[16] Jimenez-Blasco D, Santofimia-Castano P, Gonzalez A, Almeida A, Bolanos JP (2015). Astrocyte NMDA receptors’ activity sustains neuronal survival through a Cdk5-Nrf2 pathway. Cell Death Differ..

[17] Bell KF (2015). Neuronal development is promoted by weakened intrinsic antioxidant defences due to epigenetic repression of Nrf2. Nat. Commun..

[18] Skibinski G (2017). Nrf2 mitigates LRRK2- and alpha-synuclein-induced neurodegeneration by modulating proteostasis. Proc. Natl Acad. Sci. USA.

[19] Rockenstein E (2002). Differential neuropathological alterations in transgenic mice expressing alpha-synuclein from the platelet-derived growth factor and Thy-1 promoters. J. Neurosci. Res..

[20] Chesselet MF (2012). A progressive mouse model of Parkinson’s disease: the Thy1-aSyn (“Line 61”) mice. Neurotherapeutics.

[21] Games D (2013). Axonopathy in an alpha-synuclein transgenic model of Lewy body disease is associated with extensive accumulation of C-terminal-truncated alpha-synuclein.. Am. J. Pathol..

[22] Kim C (2018). Immunotherapy targeting toll-like receptor 2 alleviates neurodegeneration in models of synucleinopathy by modulating alpha-synuclein transmission and neuroinflammation. Mol. Neurodegener..

[23] Vargas MR, Johnson DA, Sirkis DW, Messing A, Johnson JA (2008). Nrf2 activation in astrocytes protects against neurodegeneration in mouse models of familial amyotrophic lateral sclerosis. J. Neurosci..

[24] Malhotra D (2010). Global mapping of binding sites for Nrf2 identifies novel targets in cell survival response through ChIP-Seq profiling and network analysis. Nucleic Acids Res..

[25] Hirotsu Y (2012). Nrf2-MafG heterodimers contribute globally to antioxidant and metabolic networks. Nucleic Acids Res..

[26] Raghunath A (2018). Antioxidant response elements: discovery, classes, regulation and potential applications. Redox Biol..

[27] Cho HY, Kleeberger SR (2020). Mitochondrial biology in airway pathogenesis and the role of NRF2. Arch. Pharm. Res..

[28] Knatko EV (2020). Downregulation of Keap1 confers features of a fasted metabolic state. iScience.

[29] Ham J, Eilers A, Whitfield J, Neame SJ, Shah B (2000). c-Jun and the transcriptional control of neuronal apoptosis. Biochem Pharm..

[30] Brown PH, Alani R, Preis LH, Szabo E, Birrer MJ (1993). Suppression of oncogene-induced transformation by a deletion mutant of c-jun. Oncogene.

[31] Scudamore O, Ciossek T (2018). Increased oxidative stress exacerbates alpha-synuclein aggregation in vivo. J. Neuropathol. Exp. Neurol..

[32] Scarlata S, Golebiewska U (2014). Linking alpha-synuclein properties with oxidation: a hypothesis on a mechanism underling cellular aggregation. J. Bioenerg. Biomembr..

[33] Yates MS (2007). Pharmacodynamic characterization of chemopreventive triterpenoids as exceptionally potent inducers of Nrf2-regulated genes. Mol. Cancer Ther..

[34] Konermann S (2015). Genome-scale transcriptional activation by an engineered CRISPR-Cas9 complex. Nature.

[35] Baxter PS, Hardingham GE (2016). Adaptive regulation of the brain’s antioxidant defences by neurons and astrocytes. Free Radic. Biol. Med.

[36] Gupta K, Chandran S, Hardingham GE (2013). Human stem cell-derived astrocytes and their application to studying Nrf2-mediated neuroprotective pathways and therapeutics in neurodegeneration. Br. J. Clin. Pharmacol..

[37] Gupta K (2012). Human embryonic stem cell derived astrocytes mediate non-cell-autonomous neuroprotection through endogenous and drug-induced mechanisms. Cell Death Differ..

[38] Vargas MR, Johnson JA (2009). The Nrf2-ARE cytoprotective pathway in astrocytes. Expert Rev. Mol. Med.

[39] Brennan MS, Matos MF, Richter KE, Li B, Scannevin RH (2017). The NRF2 transcriptional target, OSGIN1, contributes to monomethyl fumarate-mediated cytoprotection in human astrocytes. Sci. Rep..

[40] Linker RA (2011). Fumaric acid esters exert neuroprotective effects in neuroinflammation via activation of the Nrf2 antioxidant pathway. Brain.

[41] Blewett MM (2016). Chemical proteomic map of dimethyl fumarate-sensitive cysteines in primary human T cells. Sci. Signal.

[42] Schulze-Topphoff U (2016). Dimethyl fumarate treatment induces adaptive and innate immune modulation independent of Nrf2. Proc. Natl Acad. Sci. USA.

[43] Hardingham GE, Lipton SA (2011). Regulation of neuronal oxidative and nitrosative stress by endogenous protective pathways and disease processes. Antioxid. Redox Signal.

[44] Bell KF, Fowler JH, Al-Mubarak B, Horsburgh K, Hardingham GE (2011). Activation of Nrf2-regulated glutathione pathway genes by ischemic preconditioning. Oxid. Med Cell Longev..

[45] Bell KF (2011). Mild oxidative stress activates Nrf2 in astrocytes, which contributes to neuroprotective ischemic preconditioning. Proc. Natl Acad. Sci. USA.

[46] Wahl AS (2009). Hypoxic/ischemic conditions induce expression of the putative pro-death gene Clca1 via activation of extrasynaptic N-methyl-D-aspartate receptors. Neuroscience.

[47] Soriano FX (2008). Induction of sulfiredoxin expression and reduction of peroxiredoxin hyperoxidation by the neuroprotective Nrf2 activator 3H-1,2-dithiole-3-thione. J. Neurochem.

[48] Kanninen K (2009). Intrahippocampal injection of a lentiviral vector expressing Nrf2 improves spatial learning in a mouse model of Alzheimer’s disease. Proc. Natl Acad. Sci. USA.

[49] Narasimhan M, Mahimainathan L, Rathinam ML, Riar AK, Henderson GI (2011). Overexpression of Nrf2 protects cerebral cortical neurons from ethanol-induced apoptotic death. Mol. Pharm..

[50] Xiong W, MacColl Garfinkel AE, Li Y, Benowitz LI, Cepko CL (2015). NRF2 promotes neuronal survival in neurodegeneration and acute nerve damage. J. Clin. Invest.

[51] Deighton RF (2014). Nrf2 target genes can be controlled by neuronal activity in the absence of Nrf2 and astrocytes. Proc. Natl Acad. Sci. USA.

[52] Lewerenz J (2014). Phosphoinositide 3-kinases upregulate system xc(-) via eukaryotic initiation factor 2alpha and activating transcription factor 4 - A pathway active in glioblastomas and epilepsy. Antioxid. Redox Signal.

[53] Bell KF, Hardingham GE (2011). The influence of synaptic activity on neuronal health. Curr. Opin. Neurobiol..

[54] Bell KF, Hardingham GE (2011). CNS peroxiredoxins and their regulation in health and disease. Antioxid. Redox Signal.

[55] Soriano FX, Papadia S, Bell KF, Hardingham GE (2009). Role of histone acetylation in the activity-dependent regulation of sulfiredoxin and sestrin 2. Epigenetics.

[56] Webb JL, Ravikumar B, Atkins J, Skepper JN, Rubinsztein DC (2003). Alpha-Synuclein is degraded by both autophagy and the proteasome. J. Biol. Chem..

[57] Rokad D (2017). Role of neurotoxicants and traumatic brain injury in alpha-synuclein protein misfolding and aggregation. Brain Res Bull..

[58] Cristovao AC (2012). NADPH oxidase 1 mediates alpha-synucleinopathy in Parkinson’s disease. J. Neurosci..

[59] Xiang W (2013). Oxidative stress-induced posttranslational modifications of alpha-synuclein: specific modification of alpha-synuclein by 4-hydroxy-2-nonenal increases dopaminergic toxicity. Mol. Cell Neurosci..

[60] Schildknecht S (2013). Oxidative and nitrative alpha-synuclein modifications and proteostatic stress: implications for disease mechanisms and interventions in synucleinopathies. J. Neurochem.

[61] Kwak MK, Wakabayashi N, Greenlaw JL, Yamamoto M, Kensler TW (2003). Antioxidants enhance mammalian proteasome expression through the Keap1-Nrf2 signaling pathway. Mol. Cell Biol..

[62] Kapeta S, Chondrogianni N, Gonos ES (2010). Nuclear erythroid factor 2-mediated proteasome activation delays senescence in human fibroblasts. J. Biol. Chem..

[63] Nadeau PJ, Charette SJ, Toledano MB, Landry J (2007). Disulfide Bond-mediated multimerization of Ask1 and its reduction by thioredoxin-1 regulate H(2)O(2)-induced c-Jun NH(2)-terminal kinase activation and apoptosis. Mol. Biol. Cell.

[64] Reisman SA (2015). Topical application of RTA 408 lotion activates Nrf2 in human skin and is well-tolerated by healthy human volunteers. BMC Dermatol.

[65] Creelan BC (2017). Safety, pharmacokinetics, and pharmacodynamics of oral omaveloxolone (RTA 408), a synthetic triterpenoid, in a first-in-human trial of patients with advanced solid tumors. Onco Targets Ther..

[66] Reisman SA (2019). Pharmacokinetics and pharmacodynamics of the novel Nrf2 activator omaveloxolone in primates. Drug Des. Devel Ther..

[67] Lynch DR (2019). Safety, pharmacodynamics, and potential benefit of omaveloxolone in Friedreich ataxia. Ann. Clin. Transl. Neurol..

[68] Madsen KL (2020). Safety and efficacy of omaveloxolone in patients with mitochondrial myopathy: MOTOR trial. Neurology.

[69] Lynch DR (2020). Safety and efficacy of omaveloxolone in Friedreich Ataxia (MOXIe Study). Ann. Neurol..

[70] Kamemura N, Oyama K, Kanemaru K, Yokoigawa K, Oyama Y (2017). Diverse cellular actions of tert-butylhydroquinone, a food additive, on rat thymocytes. Toxicol. Res (Camb.).

[71] Gharavi N, Haggarty S, El-Kadi AO (2007). Chemoprotective and carcinogenic effects of tert-butylhydroquinone and its metabolites. Curr. Drug Metab..

[72] Braeuning A, Vetter S, Orsetti S, Schwarz M (2012). Paradoxical cytotoxicity of tert-butylhydroquinone in vitro: What kills the untreated cells?. Arch. Toxicol..

[73] Eskandani M, Hamishehkar H, Ezzati Nazhad Dolatabadi J (2014). Cytotoxicity and DNA damage properties of tert-butylhydroquinone (TBHQ) food additive. Food Chem..

[74] Satoh T, Lipton S (2017). Recent advances in understanding NRF2 as a druggable target: development of pro-electrophilic and non-covalent NRF2 activators to overcome systemic side effects of electrophilic drugs like dimethyl fumarate. F1000Res.

[75] Liu XS (2018). Rescue of fragile X syndrome neurons by DNA methylation editing of the FMR1 gene. Cell.

[76] Shao J (2018). Synthetic far-red light-mediated CRISPR-dCas9 device for inducing functional neuronal differentiation. Proc. Natl Acad. Sci. USA.

[77] Xu X, Qi LS (2019). A CRISPR-dCas toolbox for genetic engineering and synthetic biology. J. Mol. Biol..

[78] Puddifoot C (2012). PGC-1alpha negatively regulates extrasynaptic NMDAR activity and excitotoxicity. J. Neurosci..

[79] Hasel P (2017). Neurons and neuronal activity control gene expression in astrocytes to regulate their development and metabolism. Nat. Commun..

[80] Chan K, Lu R, Chang JC, Kan YW (1996). NRF2, a member of the NFE2 family of transcription factors, is not essential for murine erythropoiesis, growth, and development. Proc. Natl Acad. Sci. USA.

[81] McKenzie GJ (2005). Nuclear Ca2+ and CaM kinase IV specify hormonal- and Notch-responsiveness. J. Neurochem.

[82] Bolte S, Cordelieres FP (2006). A guided tour into subcellular colocalization analysis in light microscopy. J. Microsc.

[83] Dobin A (2013). STAR: ultrafast universal RNA-seq aligner. Bioinformatics.

[84] Liao Y, Smyth GK, Shi W (2014). FeatureCounts: an efficient general purpose program for assigning sequence reads to genomic features. Bioinformatics.

[85] Yates A (2016). Ensembl 2016. Nucleic Acids Res..

[86] Love MI, Huber W, Anders S (2014). Moderated estimation of fold change and dispersion for RNA-seq data with DESeq2. Genome Biol..

[87] Baxter PS (2015). Synaptic NMDA receptor activity is coupled to the transcriptional control of the glutathione system. Nat. Commun..

