# Supplementary material for: Targeted de-repression of neuronal Nrf2 inhibits α-synuclein accumulation
Source: Cell Death Dis. 2021 Feb 26;12(2):218. doi: 10.1038/s41419-021-03507-z (PMC7910424; doi:10.1038/s41419-021-03507-z)
Supplement: Supplementary file 1 — Supplemental Figures and Legends [file 41419_2021_3507_MOESM1_ESM.pdf]

Supplemental Figures

Figure S1

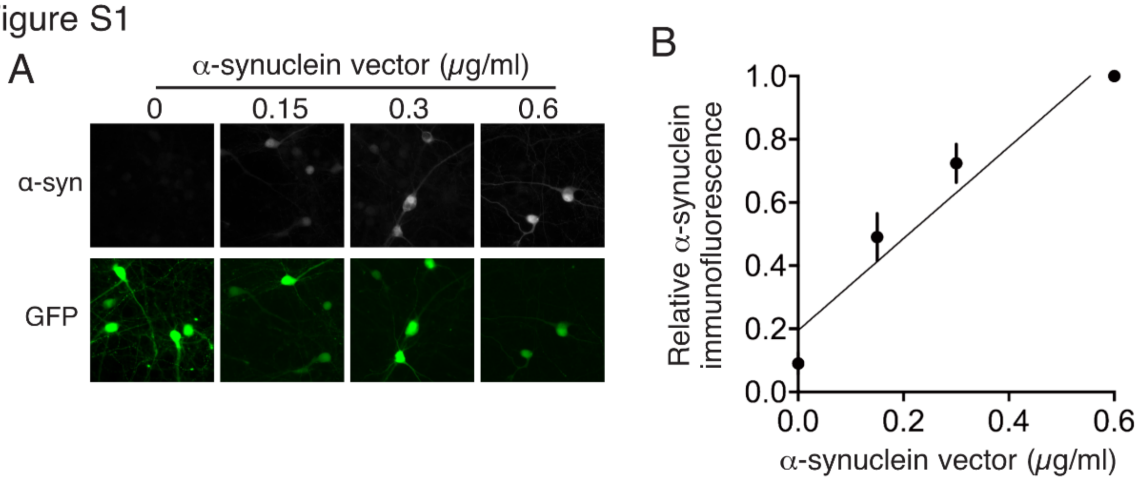

Supplemental Figure S1. A,B) Neurons were transfected with 0.15 μg/ml eGFP vector, plus the indicated concentration of α-synuclein vector. 5d post-transfection, cells were fixed and α-synuclein quantified my immunofluorescence (see Methods). 98-177 cells were analysed per condition across n=4 independent experiments

Figure S2

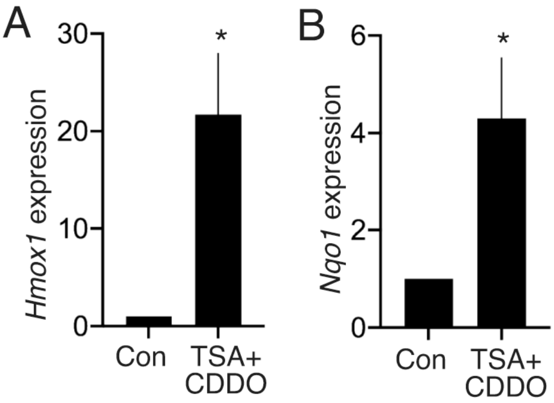

Supplemental Figure S2. A,B) Neurons were treated where indicated with TSA, followed by $CDDO^{TFEA}$ as per Fig. 3j, after which RNA was extracted and the indicated genes analysed by qPCR. P=0.0077 (A), 0.023 (B), 2-tailed t-test (n=6).
